# Efficacy of Cognition Support Formula® on cognitive function in older adults with subjective cognitive impairment: a protocol for a 26-week, randomised, double-blind, placebo-controlled trial

**DOI:** 10.1186/s13063-019-3431-3

**Published:** 2019-06-10

**Authors:** Adele E. Cave, Dennis H. Chang, Gerald W. Münch, Genevieve Z. Steiner

**Affiliations:** 1 NICM Health Research Institute, Western Sydney University, Locked Bag 1797, Penrith, NSW 2751 Australia; 2 School of Medicine, Western Sydney University, Penrith, NSW 2751 Australia; 3 Translational Health Research Institute (THRI), Western Sydney University, Locked Bag 1797, Penrith, NSW 2751 Australia

**Keywords:** Subjective cognitive impairment (SCI), Herbal medicine, Cognition Support Formula®, CogState®, EEG, Randomised controlled trial

## Abstract

**Background:**

Due to an ageing population in Australia there has been an increase in the number of older adults with subjective cognitive impairment (SCI), a self-reported decline in cognitive function associated with an increased risk of mild cognitive impairment and dementia. There is no current, recommended treatment for SCI; therefore, the effectiveness of a supplement approved by the Therapeutic Goods Association that has the potential to enhance cognitive function in an at-risk cohort should be tested. The primary aim of this proposed research is to determine the efficacy of 6 months of treatment with BioCeuticals Cognition Support Formula® (containing *Bacopa monniera* (brahmi), *Ginkgo biloba, Panax ginseng* and alpha-lipoic acid) on cognition in older adults with SCI (utilising the CogState® one card learning and identification tests as co-primary outcome measures of visual short-term memory and attention; mean speed (ms), accuracy (%), and total number of hits, misses, and anticipations) compared with placebo. The secondary aims are to assess an improvement in other cognitive domains (executive functioning, processing speed, and working memory), evaluate safety, adverse effects, and determine efficacy on mood, fatigue, and neurocognition. It is expected that improvements across the study timepoints in the co-primary outcomes in the active treatment group (compared with placebo) will be evident.

**Method:**

One-hundred and twenty participants will be recruited for the randomised, double-blind, placebo-controlled study. Participants will be randomly assigned to one of the treatment groups (active or placebo) at a 1:1 ratio, and will be required to complete a series of cognitive (using CogState®), mood (using the Depression, Anxiety, Stress Scale (DASS-42) and Short Health Anxiety Inventory (SHAI)), and fatigue (using the Functional Assessment of Chronic Illness Therapy Fatigue Scale (FACIT-F)) tasks at baseline (0 months), the midpoint (3 months), and the endpoint (6 months). These tasks will be evaluated between timepoints (baseline vs. midpoint, midpoint vs. endpoint, and baseline vs. endpoint). Neurocognition will be measured by electroencephalography at baseline and at the endpoint in half of the participants. Adverse effects will be documented over the 6-month trial period.

**Discussion:**

This is the first study to test the efficacy of Cognition Support Formula® on cognition in older adults with SCI. As people with SCI have an increased risk of dementia, and there are limited treatments options for this population, it is important to assess a supplement that has the potential to enhance cognitive function.

**Trial registration:**

Universal Trial Number (UTN), U1111–1196-9548. Australian New Zealand Clinical Trials Registry, ACTRN12617000945325. Registered on 30 June 2017.

**Electronic supplementary material:**

The online version of this article (10.1186/s13063-019-3431-3) contains supplementary material, which is available to authorized users.

## Background

### Subjective cognitive impairment

Subjective cognitive impairment (SCI) is a concern an individual has about a decline in their cognitive functioning, particularly within the memory domain [1–3]. Although most subjective reports of cognitive decline are about memory, SCI can also relate to perceived changes in attention, executive functioning, and language. Furthermore, SCI is correlated with high scores of depression and anxiety and low future cognitive performance [4]. In contrast to normal ageing, SCI is evidenced by a future objective decline in cognition over a shorter period of time than what is expected for age-related cognitive decline [5]. Hence, SCI is related to a significant future decline in cognition, rather than aged-related deficits in current memory [6].

SCI has been found to be associated with an increased risk of mild cognitive impairment (MCI) and Alzheimer’s disease (AD), the most common form of sporadic late-onset dementia [2, 7, 8]. A longitudinal study of older adults found that 54% of older adults with SCI show a cognitive decline over a 7-year follow-up period compared with only 15% with no SCI [9]. Approximately 79% of those people with SCI developed MCI, and 21% developed AD over 7 years [9], representing a significantly increased risk of future decline for this population. It is important not to ignore reports of cognitive impairment by older adults, as the literature suggests these could be early signs for the future development of AD and MCI [10].

Due to an ageing population in Australia, cognitive impairment is becoming more prevalent. The estimated global prevalence of SCI ranges between 25 and 50% in older adults [11]. In terms of prevalence between sexes, older females more often report SCI than older males [12]. This was evidenced in a study of older adults 64 years of age and above, where 35.2% of females compared with 28% of males in the study reported SCI [12]. In addition, older adults with higher levels of education compared with older adults with lower levels of education are more likely to report cognitive impairment as they are thought to have more insight into the changes in their own cognitive functioning [12].

Older adults reporting SCI are more likely to have heightened levels of anxiety and depression, which can further impact on cognitive performance and increase dementia risk, and vice versa [6, 13]. Furthermore, evidence of heightened fatigue and low energy levels have been found to be associated with poor cognitive performance in older adults with SCI [5].

### Current treatments for SCI

Current treatments for older adults with SCI are limited, with most published clinical trials utilising nutraceuticals including soybean-derived phosphatidylserine (SB-PS) and multivitamins containing extracts of *Bacopa monniera* (whole plant) and *Ginkgo biloba* leaf extract [14, 15]. These studies have documented small effects in terms of improvements in cognitive tasks (working memory, recognition memory, and recall) where short trial periods were utilised (12 weeks [14] and 16 weeks [15]). Unfortunately, there is no evidence to determine whether these effects were stable over the trial period (possibly a significant increase, then a tapering off), or whether the cognitive improvements occurred shortly after treatment, or towards the end of the trial period. A longer trial period of 6 months that includes midpoint testing is required to clarify the stability and longevity of any changes. This would prove most fruitful in determining whether such a treatment could manage SCI in the long term.

### Cognition Support Formula®

Cognition Support Formula® (BioCeuticals Pty Ltd) is a herbal and nutritional supplement commercially available over the counter in select pharmacies and via practitioners (e.g. naturopaths). Cognition Support Formula® incorporates extracts of four different ingredients into one formula—*B. monniera* (brahmi), *G. biloba*, *Panax ginseng*, and alpha-lipoic acid (ALA)—and is designed to enhance cognitive functioning and energy levels in older adults. Each of these active components will be reviewed briefly below, with evidence of their efficacy in improving cognition in older adults highlighted.

### *Bacopa monniera* (brahmi)

*B. monniera* (brahmi) is an aquatic plant utilised in the traditional Ayurvedic medical system in India to enhance memory and intellectual functioning and to promote longevity [16]. The active components of brahmi are identified as bacosides A and B [17]. Brahmi may be neuroprotective and memory enhancing, and have antioxidant effects [18]. The active bacosides are thought to increase cerebral blood flow and neurotransmitter modulation (acetylcholine, 5-hydroxytryptamine, and dopamine) [18]. Brahmi has been found to significantly improve memory acquisition and retention in immediate and delayed recall task in 36 older adults ingesting one 300 mg tablet/day for 12 weeks (compared with *n* = 45 in the control group) [16].

### *Ginkgo biloba* (ginkgo)

*G. biloba* (ginkgo) is a natural extract derived from the leaf of the Chinese ginkgo tree. Its bioactive constituents (flavonoids and terpenoids) are thought to have strong antioxidant properties, which assist in slowing down the progression of age-related diseases [19]. These components have been found to increase the blood supply by dilating blood vessels and reducing blood viscosity. The standardised extracts of ginkgo leaf (EGb 761) have been shown to increase positive mood, energy, and cognition [1, 19]. One study of 241 older adults showed an increase in scores on the Benton Visual Retention Test-Revised in two active groups (low-dose group, 1.9 ml undiluted ginkgo extract three times daily with a placebo; high-dose group, 1.9 ml of undiluted extract three times daily) for 24 weeks compared with a placebo [1].

### *Panax ginseng*

*P. ginseng*, or Korean ginseng, is an extract from the Araliaceae family of plants [20]. The active component is ginsenosides from three different groups: protopanaxadiol, protopanaxatriol, and oleanolic acid. The mechanism of action of ginseng is thought to be within the central nervous system with the modulation of neurotransmitter systems involved in the mediation of memory and cognition, including GABA, glutamate, dopamine, and serotonin [21]. Additionally, ginseng has been found to upregulate key neurotrophins involved in neuroplasticity including brain-derived neurotropic factor (BDNF) [22]. For a detailed review on the molecular mechanisms of ginseng compounds see Lü et al. [23].

In combination with ginkgo, ginseng has been shown to improve aspects of working memory and improved attention in younger adults [20]. Additionally, Sünram-Lea and colleagues found improvements in 15 participants regarding their processing speed and in episodic memory with an acute ingestion of 400 mg ginseng compared with a placebo group of the same sample size [24]. This outcome is of particular interest since episodic memory is a key deficit in adults with AD.

### Alpha-lipoic acid

ALA, a naturally occurring fatty acid, is a cofactor of some mitochondrial enzymes. ALA is a powerful nutrient that is able to recycle redox-active antioxidant vitamins such as E and C [25]. Mechanisms of action include an influence on neuronal energy production (reactivation of enzymes involved in glucose metabolism and glucose transport, i.e. a blood glucose lowering effect) and anti-oxidative and anti-inflammatory properties (downregulation of chronic inflammatory processes) [26].

In the literature, ALA has been found to stabilise cognitive functioning in older adults with AD [27]. Nine participants with probable AD took part in a non-randomised open-label study for an average of 337 days where they ingested 600 mg ALA daily [27]. Mini-Mental State Examination (MMSE) and the Alzheimer’s Disease Assessment Scale-cognitive (ADAS-cog) subscale scores were stable (i.e. no decline) for the trial duration.

### Adverse effects

The proposed study detailed in this protocol is a clinical trial which will document the safety and any adverse effects of Cognition Support Formula® which uses a combination of the four ingredients. Separately, however, minor adverse effects have been reported for brahmi, ginseng, and ALA. Morgan and Stevens [16] noted minor gastrointestinal upset in participants receiving brahmi treatment compared with placebo. Similarly, over a 4-week treatment period ingesting 1000 mg ginseng per day, mild gastrointestinal upset and insomnia were evidenced compared with placebo [28]. Gastrointestinal upset has been recorded as an adverse effect after ingesting ginkgo; however, this effect was not statistically significant compared with ingestion of placebo [1, 19].

Drug interactions have been reported for cyclophosphamide and levothyroxine, and these should be avoided when taking ALA due to their possible adverse effects. ALA can combine with these medications, which may lower blood sugar levels and thyroid hormone levels [29].

### Rationale and aims

Currently, there are limited treatment options for an older population reporting SCI. Due to this limitation, and the increased risk of developing MCI and dementia, the efficacy of any intervention claiming potential cognitive enhancement should be tested. As Cognition Support Formula® is a commercially available and listed medicine approved by the Therapeutic Goods Association (TGA) and it has the potential to improve cognition, its efficacy and safety in a cohort of older adults with SCI requires testing.

The primary aim is to determine the efficacy of 6 months of treatment with Cognition Support Formula® to improve cognition in older adults with SCI as measured by the one card learning test and the identification test (from the CogState® Pre-clinical Alzheimer’s Battery). These tests measure visual short-term memory and attention and will be considered as co-primary outcome measures as they are the most common areas of cognitive decline cited in the current literature for this population, and from the observations made in our laboratory [1–3]. The secondary aims are: 1) to assess an improvement in the cognitive domains of processing speed and working memory by utilising the detection test and the one back task (derived from the CogState® Pre-clinical Alzheimer’s Battery) and, in addition, executive functioning will be measured by the Groton Maze learning test (an additional CogState® test); 2) to assess the safety and adverse effects of Cognition Support Formula® compared with placebo; 3) to assess the efficacy of 6 months of treatment with Cognition Support Formula® compared with placebo on mood, as measured by the Depression, Anxiety, Stress Scale (DASS-42) and the Short Health Anxiety Inventory (SHAI), and fatigue and energy levels, as measured by the Functional Assessment of Chronic Illness Therapy Fatigue Scale (FACIT-F); and 4) to assess the efficacy of 6 months of treatment with Cognition Support Formula® on neurocognition compared with placebo by utilising psychophysiological measures (electroencephalography (EEG)) at baseline and at the endpoint.

In line with previous research examining individual constituents [1, 14, 16–25], we hypothesise that a 6-month dose of Cognition Support Formula® will improve cognitive functioning with minimal adverse effects in older adults with SCI compared with placebo. Due to limited literature in this area, exploratory analyses will be conducted in the domains of mood, fatigue, and neurocognition.

## Methods

### Trial design and setting

A randomised, double-blind, placebo-controlled 6-month trial will test the efficacy and safety of Cognition Support Formula® on cognition, mood, fatigue, and electrophysiology in older adults with SCI. The current study protocol (version 6) was designed in accordance with the SPIRIT guidelines [30] (see Additional file 1) and Good Clinical Practice guidelines (1996). Any changes to the study protocol will be communicated with the study investigative team, the Australian New Zealand Clinical Trials Registry (ANZCTR), and the Western Sydney University Human Research Ethics Committee. The study is being conducted in the NICM Clinical Laboratory and HEADBOX Laboratory at Western Sydney University, Campbelltown campus.

### Intervention

#### Active

Cognition Support Formula® contains clinically trialled ingredients to assist learning and memory. The composition of the formula components can be found in Table 1. The daily dose will be one tablet, twice daily (morning and night), for 6 months. Cognition Support Formula® is a dietary supplement made by BioCeuticals Pty Ltd, and is approved as a listed medicine by the TGA.

**Table 1:** Composition of Cognition Support Formula® components

*Bacopa monniera* (bacopa) whole plant (equivalent to bacosides A)	4.00 g 90.00 mg
*Ginkgo biloba* (ginkgo) leaf (equivalent to ginkgo flavonglycosides) (equivalent to ginkgolides and bilobalide)	3.00 g 16.02 mg 4.02 mg
*Panax ginseng* (Korean ginseng) root (equivalent to ginsenosides Rg1, Re, Rf, Rg2, Rb1, Rb2, Rc, Rd)	212.00 mg 4.25 mg
R, S-alpha-lipoic acid	300.00 mg

#### Placebo

The placebo tablet contains an inert substance, which is matched for colour, smell, and taste to that of Cognition Support Formula®. The dose will be one tablet, twice daily (morning and night), for 6 months. The composition of components contained within the placebo tablet can be found in Table 2.

**Table 2:** Composition of placebo components

Calcium hydrogen phosphate	782.00 mg
Microcrystalline cellulose	532.00 mg
Silica—colloidal anhydrous	12.00 mg
Magnesium stearate	12.00 mg

### Inclusion and exclusion criteria

The inclusion criteria for the proposed study are:

- ≥ 60 years of age
- Evidence of SCI as measured by:
  - ◦ Answering ‘yes’ to any of the following questions:
    - ▪ Do you feel your memory and thinking is getting worse?
    - ▪ Do you feel your memory and thinking has become worse over the past 2–3 years?
    - ▪ Are you concerned about your decline in memory and thinking?
  - ◦ No self-reported diagnosis of dementia or MCI
  - ◦ Scoring ≥ 23 on the Montreal Cognitive Assessment (MoCA) (as there is no standardised measure for SCI, a score of ≥ 23 will be utilised as a cut-off for SCI in line with previous literature [31, 32]
- Normal vision or vision corrected to normal
- Normal hearing or aided hearing

The exclusion criteria for the proposed study are:

- Severe depression, as measured by scoring > 19 on the 30-item Geriatric Depression Scale (GDS)
- Use of psychoactive medications or excessive alcohol intake (a maximum of 14 standard drinks per week) as recommended by the Australian Department of Health
- No caffeine 2 h prior to testing
- Non-smokers
- No history of seizures or head injury (with loss of consciousness)
- Allergy to the study drug ingredients (ginseng, ginkgo, brahmi, or ALA)
- Participants previously taking ginseng, ginkgo, brahmi, or ALA (either separately or as a component of a supplement) are excluded from participation unless they have discontinued using these ingredients 8 weeks prior to testing
- Left-handedness (only for EEG testing [33])
- Individuals with type 2 diabetes with a high fasting glucose level at baseline (> 8 mmol/L, according to Diabetes Australia) or those experiencing complications associated with their diabetic condition
- Diagnosed psychiatric disorders including bipolar disorder, schizophrenia, personality disorders, drug and alcohol dependence, or substance abuse disorders
- Presence or history of severe renal and hepatic disorders
- High dependence on medical care (including medications) due to past or current medical conditions, for example cancer
- Participants using cyclophosphamide and levothyroxine

### Recruitment and consent

Recruitment will be conducted by several methods, including advertisements in local newspapers, flyers at local community and residential aged-care facilities, internet sites (Western Sydney University and NICM websites), NICM and HEADBOX Lab newsletters, and social media promotion through Facebook and Twitter. In addition, participants will be referred from other trials conducted at HEADBOX Lab including a randomised, double-blind, placebo-controlled 12-week trial of Sailuotong (SLT) for mild cognitive impairment (Western Sydney University Human Research Ethics Committee Approval: H11878). Retention will be facilitated by regular contact during the clinical trial for screening, appointment reminders, and the three testing sessions (baseline, midpoint, and endpoint). Participants will provide informed verbal consent via telephone and written informed consent at the time of face-to-face screening to a member of the research team.

### Ethical guidelines

This research will be conducted in accordance with the International Ethical Guidelines for Biomedical Research Involving Human Subjects prepared by the Council for International Organisations of Medical Sciences (CIOMS) in collaboration with the World Health Organization (WHO), and the National Health and Medical Research Council (NHMRC).

### Privacy and data management

In accordance with ethical guidelines, the anonymity, confidentiality, and privacy of participants will be protected. All electronic data will be password protected. Hardcopy source data records will be kept in a locked cabinet in the office of the principal investigator during data collection and at completion of the study for an indefinite period of time. All publication material will refer to general trial results as aggregate data and no individual participant names or identifying information will be released. Participants are welcome to view their personal data at any time during the trial.

As this is an investigator-led study with a small sample size, a data management committee and auditing process is not required. Quality control, decisions pertaining to amendments of the protocol, coordination of the trial, and development of the secure database and trial forms will be completed by the research team. Data will be stored within the secure REDCap system (version 8.5.7) and coded utilising IBM SPSS statistics software (version 23).

### Sample size for co-primary and secondary outcome measures

This is the first study to utilise Cognition Support Formula® as a treatment for SCI, and thus there are limited comparable studies on which to base a sample size estimation. The most comparable study is by Lim et al. [34], who utilised a similar population (healthy older adults vs. people with MCI vs. AD) and a comparable measure to the current study as outlined in this protocol (CogState® battery). In order to see an improvement in performance in one test of the CogState battery® (group × time interaction) an effect size of Cohen’s *d* = .40 (i.e. Cohen’s *f* = .20, as *d* = 2*f*) at α = .05 was utilised [34].

To detect this effect size of Cohen’s *f* = .20 at α = .05 and 95% power, 100 participants across two groups are required. In order to account for a typical 20% drop-out, a total of 120 participants will need to be recruited (60 participants in the treatment group, and 60 in the placebo group). If the participation rate drops below the estimated number at any point during the course of the study, attempts to recruit additional participants will be made. Sample size may be re-estimated mid-course into the study to ensure adequate power. In this case, data will be unblinded by an objective third party to evaluate the treatment-effect size and power to assist with any potential sample size re-estimation.

### Sample size for EEG sub-study

Most EEG studies do not utilise effect size calculations to determine sample size [35]. It is more important to determine signal-to-noise ratio in event-related potential (ERP) studies to ensure that the stimulus-evoked activity can be differentiated from the ongoing background EEG. Therefore, to obtain a meaningful EEG sub-study sample size (greater than 20 participants across both groups), the total sample size for EEG testing is 64 participants; 32 derived from the treatment group and 32 from the placebo group.

### Randomisation, allocation concealment, compliance and adverse effects

Participants will be randomly allocated at a 1:1 ratio to either the treatment or placebo group using a permuted block allocation strategy. Computerised randomisation and allocation will be conducted using a unique random number generator in Microsoft Excel by a university staff member external to the research team. This external staff member sent the random sequence directly to the trial drug manufacturer, with participant IDs and unique batch codes to conceal the sequence. This is a double-blind trial, so both participants and the research team are blinded to treatment allocation until study completion. After eligibility is confirmed, participants will then be allocated to the next participant ID/numbered box of product by a member of the research team. Compliance will be assessed by advising participants to return any unused medication at the midpoint (3 months) and endpoint (6 months), and by completion of a diary by participants noting medication intake and adverse effects.

To monitor safety, participants will be referred to a local commercial laboratory (Laverty) prior to baseline and the endpoint testing sessions. Standard blood safety tests (full blood count, fasting blood glucose, liver and renal function tests) will be carried out. If participants receive an abnormal blood safety test result at baseline, they will not be enrolled in the study and will be encouraged to follow up their results with their regular health professional. Adverse events will be closely monitored throughout the course of the study.

In the case of a serious adverse event, and if the study drug is suspected as a potential cause and identifying treatment allocation is essential to the participant receiving appropriate medical care, unblinding of that participant only will occur. This will be done by the university staff member external to the research team who generated the computerised random sequence allocation.

At the study midpoint, endpoint, and follow-up (7 months; 4 weeks after the endpoint) participants will be asked to which treatment condition they think that they have been allocated. Responses of Cognition Support Formula®, placebo, or unknown will be recorded. Responses will be checked against the condition allocated at the end of the trial after un-blinding. Participants who exit the trial early will also be contacted via telephone and asked to complete a trial exit interview pertaining to their experiences of the trial.

### Testing schedule

A detailed testing schedule of the trial protocol is depicted in Table 3.

**Table 3:** Testing schedule of participants

Testing schedule	Task	Telephone screen	Face-to-face screen	Baseline(0 months)	Midpoint(3 months)	Endpoint(6 months)	Follow-up 4 weeks after endpoint(7 months)
Screening assessment	TICS-M	X					
	General medical history		X				
	MoCA		X				
	GDS		X				
	RAVLT		X				
CogState® battery	Identification task^a^			X	X	X	
	One card learning test^a^			X	X	X	
	Detection test^b^			X	X	X	
	One back test^b^			X	X	X	
CogState® additional test	Groton Maze learning test^b^			X	X	X	
Mood and fatigue outcome measures	DASS-42^b^			X	X	X	
	SHAI^b^			X	X	X	
	FACIT-F^b^			X	X	X	
Electrophysiological tasks	Word list immediate and delayed recall tasks^c^			X		X	
	Resting-state EEG^c^			X		X	
	Equiprobable Go/NoGo^c^			X		X	
Safety and compliance measures	FBC, E/LFT (bloods)^b^			X		X	
	Adverse effects^b^				X	X	X
	Compliance and blinding				X	X	X

^a^Co-primary outcome measures

^b^Secondary outcome measures

^c^EEG tasks will be completed by a subset of participants

*DASS-42* Depression, Anxiety, Stress Scale, *EEG* electroencephalography, *E/LFT* electrolyte and liver function test, *FACIT-F* Functional Assessment of Chronic Illness Therapy Fatigue Scale, *FBC* full blood count, *GDS* Geriatric Depression Scale, *MoCA* Montreal Cognitive Assessment, *RAVLT* Rey Auditory Verbal Learning Test, *SHAI* Short Health Anxiety Inventory, *TICS-M* modified telephone interview for cognitive status

### Study procedures and outcomes

**Fig. 1:**
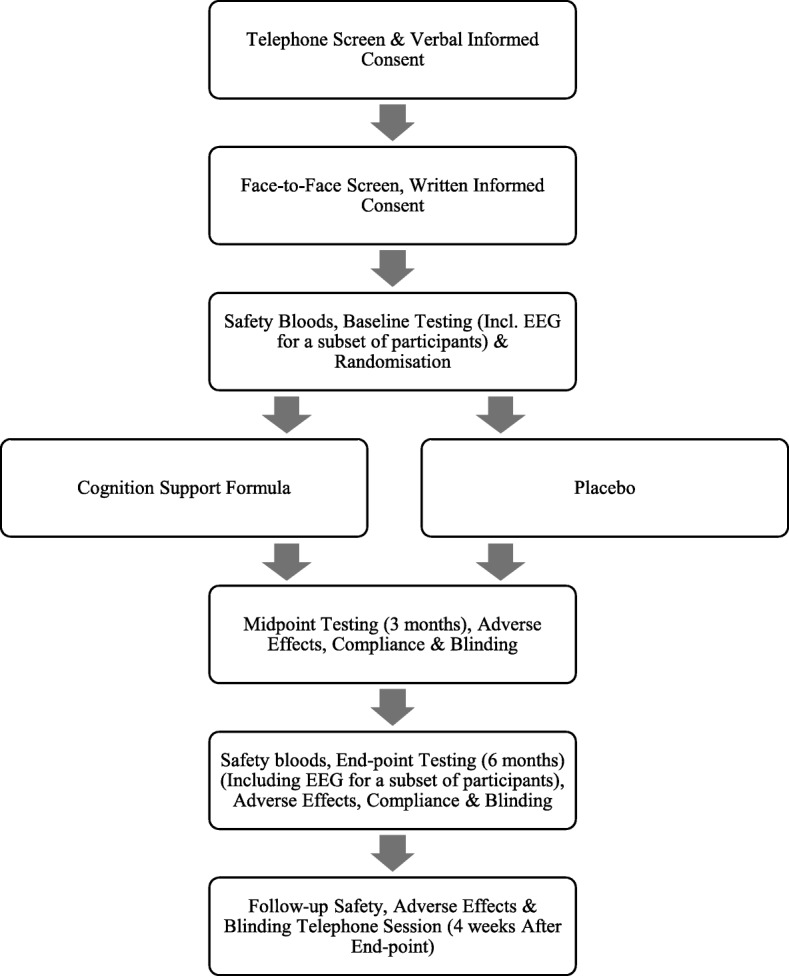
Flow diagram of participants in the proposed clinical trial

A flow diagram of the participants for the proposed clinical trial is depicted in Fig. 1.

### Screening

Telephone screening will be conducted with participants to determine their eligibility for the study. Participants will complete a modified version of the TICS-M, a validated screening tool for cognitive impairment [36], and will answer questions pertaining to the inclusion and exclusion criteria listed previously.

If successful, participants will be invited to attend the NICM Health Research Institute Clinical Research Laboratory and HEADBOX Laboratory at Western Sydney University for a face-to-face screening session. Participants will complete the GDS, Rey Auditory Verbal Learning Test (RAVLT), a general health questionnaire, and the MoCA.

### Cognitive functioning evaluation

Participants will complete a number of computerised and pen and paper cognitive tests during the trial. Table 3 lists the cognitive, mood, and fatigue measures that will be utilised in this study. Further detail of these measures are given below.

The CogState® pre-clinical Alzheimer’s Battery of tests will be utilised to measure baseline, 3-month and 6-month changes in cognition. In addition to the battery, the Groton Maze learning test (CogState® test) will be completed by participants. CogState® is a computerised cognitive test battery that has excellent test-retest reliability ranging from .76–.93 (intra-class correlation coefficient) [34]. Participants will complete a practice trial prior to the main trial, in accordance with previous literature [34, 37]. There are no known practice effects associated with completing the practice. Practice trials will help to provide a ‘true baseline’ measure as errors are generally made during the first instance of completing the task. Participants will become familiar with the tasks during the practice trials, and this will therefore ensure equity in the measurement of task outcomes between the testing timepoints. The CogState® battery will be administered at baseline (0 months), the midpoint (3 months), and the endpoint (6 months), again with no known practice effects between time intervals. Furthermore, this cognitive testing method enables investigation into multiple areas of cognition including processing speed, attention, short-term memory, working memory, and executive function. The co-primary outcomes (one card learning and identification tests) measure visual short-term memory and attention, while the secondary outcomes (Groton Maze learning test, detection, and one back tests) measure executive functioning, processing speed, and working memory, respectively.

The DASS is a 42-item self-report instrument designed to measure the three related negative emotional states of depression, anxiety, and stress. Participants who consistently score higher than the normal range for depression (0–9), anxiety (0–7), or stress (0–14) (during baseline and midpoint sessions) will be advised to speak to their general practitioner. There is an expectation that participants will score higher in each of the three domains compared with a population without SCI [4].

The SHAI is a shortened (18-item) measure of participants’ anxiety pertaining to their health. The highest total score is 52, indicating the highest level of health anxiety. Participants will complete the SHAI at baseline, the midpoint, and the endpoint. A comparison from baseline to endpoint will be made between treatment groups (active and placebo) to determine if health anxiety has decreased with the use of Cognition Support Formula® or placebo.

The FACIT-F is a self-report questionnaire utilised to determine a participant’s level of fatigue during their usual daily activities over the past 7 days. The FACIT-F is a 13-item questionnaire measuring the level of fatigue on a four-point Likert scale. The FACIT-F will be utilised to determine if fatigue and energy levels improve with the use of Cognition Support Formula®.

### EEG protocol

EEG is an objective and non-invasive method of measuring neuronal activity. Utilisation of EEG in the proposed study will complement the cognitive measures previously outlined. Potential changes in participants’ resting EEG and task-related EEG from baseline to endpoint will be investigated. These changes include short- and long-term episodic memory encoding and retrieval, arousal, response activation, inhibition, and discrimination. Additionally, differences in the above-mentioned EEG methods between the treatment and placebo groups will be investigated.

### Statistical analyses and dissemination

For each participant in each group and at each timepoint the following data from the primary and secondary outcome measures will be assessed. For the CogState® detection, identification, one card learning, and one back tests, we will assess mean speed (ms), accuracy (%), and total number of hits, misses, and anticipations; for the Groton Maze test, we will assess the total number of errors; and for the mood (DASS-42, SHAI) and fatigue (FACIT-F) outcomes, we will assess subscale and composite scores. CogState® data that are not normally distributed will be transformed using logarithmic base 10 transformations. Generalised linear mixed models with a between-subject factor of group (treatment, placebo) and within-subject factor of time (baseline, midpoint, endpoint) will be conducted to assess differences in continuous co-primary and secondary outcome measures. Planned simple contrasts will be conducted to assess all timepoints (baseline vs. midpoint, midpoint vs. endpoint, and baseline vs. endpoint). To handle any missing data, analysis of last observation carried forward will be undertaken within the intention-to-treat analysis model. The premise of the intention-to-treat model includes all participants in the final analysis even if non-compliance, withdrawal, or deviation from the protocol occurs [38]. Due to the nature of drop-outs in clinical trials, this analysis will be carried out on all five primary and secondary outcome measures of cognition and secondary outcomes of mood (DASS and SHAI) and fatigue (FACIT-F).

All tests will be two-tailed, α = .05. MANOVAs and Pearson’s correlations may also be utilised for assessment of psychophysiological data depending on the outcome measures assessed. All data will be analysed using SPSS. Results from the trial will be presented publicly to the community through peer-reviewed journals and scientific conference presentations. Additionally, individual trial outcomes will be forwarded to participants, with their consent.

## Discussion

This article presents the rationale and methods for a clinical trial on the effectiveness of Cognition Support Formula® on subjective cognitive impairment in older adults. Currently there are limited treatments available for older adults reporting SCI who are at risk of developing AD and MCI; therefore, it is important to test the efficacy of a herbal and nutritional supplement that promotes improvements in cognitive health.

While the limited current treatments have shown improvements in cognition [14, 15], the present study will consist of a longer treatment period and provide an in-depth insight into the cognitive and neuronal effects of Cognition Support Formula®. By utilising a 6-month trial period (with baseline and midpoint testing), there will be adequate coverage to obtain stable outcome measures and possibly larger changes in cognitive functioning. Furthermore, the intervals (0, 3, 6 months) will assist in better determining the effectiveness of intermediate and long-term use of the supplement. Additionally, these comparisons will also help to generalise our results to other studies that have used shorter trial periods (e.g. 12 weeks).

Each of the individual components of the formula has been found to increase cognition in older adults with cognitive impairment [1, 14, 16–25]. If results are shown to be positive, this study will lend support to the use of the Cognition Support Formula® as a complementary treatment for SCI.

This study is the first to determine the efficacy and safety of Cognition Support Formula®, a TGA-approved listed medicine for older adults with SCI. It will provide information pertaining to healthy ageing and cognitive decline, and may provide support for the use of Cognition Support Formula® in older adults with SCI. Overall, outcomes from this study will benefit the wider population, especially older adults seeking an understanding of cognitive decline and potential treatments.

## Trial status

Recruitment began in September 2017 and is estimated to conclude late in 2019.

## Additional file

Additional file 1: SPIRIT 2013 checklist: recommended items to address in a clinical trial protocol and related documents. (DOC 131 kb)

## Data Availability

Not applicable.

## Abbreviations

AD: Alzheimer’s disease
ALA: Alpha-lipoic acid
DASS-42: Depression, Anxiety, Stress Scale
EEG: Electroencephalography
FACIT-F: Functional Assessment of Chronic Illness Therapy Fatigue Scale
GDS: Geriatric Depression Scale
MCI: Mild cognitive impairment
MoCA: Montreal Cognitive Assessment
RAVLT: Rey Auditory Verbal Learning Test
SCI: Subjective cognitive impairment
SHAI: Short Health Anxiety Inventory
TGA: Therapeutic Goods Association

## References

[1] Brautigam MRH (1998). Treatment of age-related memory complaints with Ginkgo biloba extract: a randomised double blind placebo-controlled study. Phytomedicine.

[2] Jessen F (2014). A conceptual framework for research on subjective cognitive decline in preclinical Alzheimer’s disease. Alzheimers Dement.

[3] Barbhaiya HC, et al. Efficacy and tolerability of BacoMind® on memory improvement in elderly participants—a double blind placebo controlled study. J Pharmacol Toxicol. 2008;3(6):425–34.

[4] Steinberg SI (2013). Subjective memory complaints, cognitive performance, and psychological factors in healthy older adults. Am J Alzheimers Dis.

[5] Langlois AS, Belleville S (2013). Subjective cognitive complaint in healthy older adults: identification of major domains and relation to objective performance. Aging Neuropsychol C.

[6] Caramelli P, Beato RG (2008). Subjective memory complaints and cognitive performance in a sample of healthy elderly. Dementia Neuropsychologia.

[7] Buckley RF (2016). A conceptualization of the utility of subjective cognitive decline in clinical trials of preclinical Alzheimer’s disease. J Mol Neurosci.

[8] Freitas S, Simões MR, Alves L, Santana I (2013). Montreal cognitive assessment: validation study for mild cognitive impairment and Alzheimer disease. Alz Dis Assoc Dis.

[9] Reisberg B, Shulma MB, Torossian C, Leng L, Zhu W (2010). Outcome over seven years of healthy adults with and without subjective cognitive impairment. Alzheimers Dement.

[10] Brodaty H (2013). Mild cognitive impairment in a community sample: the Sydney Memory and Ageing Study. Alzheimers Dement.

[11] Jonker C, Geerlings MI, Schmand B (2000). Are memory complaints predictive for Dementia? A review of clinical and population-based studies. Int J Geriatr Psych.

[12] Montejo P, Montenegro M, Fernández MA, Maestú F (2011). Subjective memory complaints in the elderly: prevalence and influence of temporal orientation, depression and quality of life in a population-based study in the city of Madrid. Aging Ment Health.

[13] Ownby RL, Crocco E, Acevedo A, John V, Loewenstein D (2006). Depression and risk for Alzheimer disease. Arch Gen Psychiat.

[14] Richter Y, Herzog Y, Lifshitz Y, Hayun R, Zchut S (2013). The effect of soybean-derived phosphatidylserine on cognitive performance in elderly with subjective memory complaints: a pilot study. Clin Interv Aging.

[15] Macpherson H, Ellis KA, Sali A (2012). Memory improvement in elderly women following 16 weeks treatment with a combined multivitamin, mineral and herbal supplement. Psychopharmacology.

[16] Morgan A, Stevens J (2010). Does Bacopa monnieri improve memory performance in older persons? Results of a randomised, placebo-controlled, double-blind trial. J Altern Complem Med.

[17] Steiner GZ, Mathersul DC, Camfield DA, McIntyre E, Sarris J (2017). Cognitive anxiolytics. Evidence-based herbal and nutritional treatments for anxiety in psychiatric disorders.

[18] Aguiar S, Borowski T (2013). Neuropharmacological review of the nootropic herb Bacopa monnieri. Rejuv Res.

[19] Birks J, Evans JG. Ginkgo biloba for cognitive impairment and dementia (Review). Cochrane DB Syst Rev. 2009;(1). 10.1002/14651858.CD003120.pub3 PMC13076002 19160216

[20] Neale C, Camfield D, Reay J, Stough C, Scholey A (2012). Cognitive effects of two nutraceuticals Ginseng and Bacopa benchmarked against modafinil: a review and comparison of effect sizes. Brit J of Clin Pharmaco.

[21] Tsang D, Yeung HW, Tso WW, Peck H (1985). Ginseng saponins: influence on neurotransmitter uptake in rat brain synaptosomes. Planta Med.

[22] Wang Y, Feng Y, Fu Q, Li L (2013). Panax notoginsenoside Rb1 ameliorates Alzheimer’s disease by upregulating brain-deprived neurotrophic factor and downregulating Tau protein expression. Exp Ther Med.

[23] Lü JM, Yao Q, Chen C (2009). Ginseng compounds: an update on their molecular mechanisms and medical applications. Curr Vasc Pharmacol.

[24] Sünram-Lea SI, Birchall RJ, Wenes KA, Petrini O (2005). The effect of acute administration of 400mg of Panax ginseng on cognitive performance and mood in healthy young volunteers. Curr Top Nutraceut R.

[25] Maczurek A (2008). Lipoic acid as an anti-inflammatory and neuroprotective treatment for Alzheimer’s disease. Adv Drug Deliv Rev.

[26] Holmquist L (2007). Lipoic acid as a novel treatment for Alzheimer’s disease and related dementias. Pharmacol Therapeut.

[27] Hager K, Marahrens A, Kenklies M, Riederer P, Münch G (2001). Alpha-lipoic acid as a new treatment option for Azheimer type dementia. Arch Gerontol Geriat.

[28] Lee NH, Yoo SR, Kim HG, Cho JH, Son CG (2012). Safety and tolerability of Panax ginseng root extract: a randomised, placebo-controlled, clinical trial in healthy Korean volunteers. J Altern Complem Med.

[29] Stargrove MB, Treasure J, McKee DL. Herb, Nutrient, and Drug Interactions. Missouri: Mosby Elsevier; 2008.

[30] Chan AW (2013). SPIRIT 2013 explanation and elaboration: guidance for protocols of clinical trials. BMJ-Brit Med J.

[31] Julayanont P, Phillips N, Chertkow H, Nasreddine ZS, Larner A (2013). The Montreal Cognitive Assessment (MoCA): concept and clinical review. Cognitive screening instruments: a practical approach.

[32] Trzepacz PT, Hochstetler H, Wang S, Walker B, Saykin AJ (2015). Relationship between the Montreal Cognitive Assessment and Mini-mental State Examination for assessment of mild cognitive impairment in older adults. BMC Geriatr.

[33] Beratis IN (2009). Effect of initiation-inhibition and handedness on the patterns of the P50 event-related potential component: a low resolution electromagnetic tomography study. Behav Brain Funct.

[34] Lim YY (2013). Three-month stability of the CogState brief battery in healthy older adults, mild cognitive impairment, and Alzheimer’s disease: results from the Australian imaging, biomarkers, and lifestyle-rate of change substudy (AIBL-ROCS). Arch Clin Neuropsych.

[35] Larson MJ, Carbine KA (2017). Sample size calculations in human electroencephalography (EEG and ERP) studies: a systematic review and recommendations for increased rigor. Int J Psychophysiol.

[36] Brandt J, Spencer M, Folstein M (1988). The telephone interview for cognitive status. Neuropsy Neuropsy Be.

[37] Maruff P (2013). Clinical utility of the CogState brief battery in identifying cognitive impairment in mild cognitive impairment and Alzheimer’s disease. BMC Pharmacol Toxico.

[38] Armour Mike, Ee Carolyn, Steiner Genevieve Z. (2018). Randomized Controlled Trials. Handbook of Research Methods in Health Social Sciences.

